# Autologous blood transfusion in trauma: a literature review

**DOI:** 10.1186/1757-7241-23-S2-A7

**Published:** 2015-09-11

**Authors:** John E McKenna

**Affiliations:** 1 Department of Anaesthesia, Royal London Hospital, London, UK

## Background

Autologous blood transfusion encompasses a variety of techniques used to recycle and reinfuse shed blood, typically lost during elective surgery. Trauma patients often experience large volume blood loss and have high requirements for allogeneic blood; autologous blood could satisfy these requirements. Despite the theoretical advantages, the technique of autologous blood transfusion has not been widely adopted for trauma patients.

## Method

A literature search between January 2000 to March 2014 of PubMed, Embase and the Cochrane collection was performed to examine the current evidence regarding the use of autologous blood transfusion in trauma.

## Results

97 full text articles were assessed. A total of 17 papers were included in the review. The quality of the evidence was generally weak. A number of themes appeared in the literature. 11 papers examined the use of cell salvage in relation to trauma surgery, orthopaedic trauma and Jehovah's Witness patients. The remaining 6 papers examined the use of autologous blood for traumatic haemothorax, paediatrics and burns.

## Discussion

There were few papers published on autologous blood transfusion in trauma. There is evidence that certain injury patterns yield higher volumes of salvageable blood, that transfusion of autologous blood reduces the requirement of allogeneic blood and that autologous blood may be the only source of transfused blood in certain environments. The evidence was not sufficiently strong to make definitive statements regarding the safety or cost effectiveness of autologous transfusion. Further study should focus on the composition of salvaged blood, clinical consequences of autologous transfusion and injury patterns that yield the greatest volume salvageable blood.

